# What is the predictive validity of clinical placement sign-off forms for medical students?

**DOI:** 10.1186/s12909-025-07237-0

**Published:** 2025-06-05

**Authors:** Patrick J McGown, Molly M Nichols, Jennifer A Forshaw, Antonia Rich, David Harrison, Celia Brown, Amir H. Sam

**Affiliations:** 1 Imperial College School of Medicine, Imperial College London, London, UK; 2 Chelsea & Westminster NHS Trust, London, UK; 3 University College London, London, UK

**Keywords:** Undergraduate, Medical education research, Clinical assessment, Written assessment, Curriculum infrastructure

## Abstract

**Background:**

Evaluation of clinical performance is essential in all medical school programmes. Students undergo multiple clinical placements in different disciplines and settings, and typically must pass an end-of-placement supervisor sign-off evaluation to progress. However, the validity of this sign-off model remains unclear. This study aims to assess the extent to which this assessment method predicts performance in summative medical school examinations.

**Methods:**

We compared summative knowledge and clinical skills examination scores with end-of-placement supervisor sign-off ratings of ‘knowledge’, ‘clinical skills’ and ‘practical skills’ for medical undergraduate students, across three clinical placements at Imperial College London, UK (n = 355). Statistical analysis for predictive validity was performed through Ordinary Least Squares regression.

**Results:**

End-of-placement supervisor ratings in hospital did not significantly predict student performance in summative knowledge tests or clinical skills assessment. ‘Knowledge’ and ‘practical skills’ ratings lacked predictive validity across all supervisors. Statistically significant predictive validity was evident for GP supervisor ratings of ‘clinical skills’ and examination scores, but the effect size was educationally insignificant (p = 0.01, r^2^ = 0.02).

**Conclusions:**

End-of-placement supervisor ratings did not demonstrate educationally significant predictive validity towards end-of-year examinations. Multi-source feedback, embedded in-placement assessment, and additional formalised supervision time in supervisors’ work schedules could be beneficial to improve the educational value for the student and the clinical placement sign-off process. Different sign-off requirements could be considered for GP and hospital settings, with tailoring of constructs to suit the clinical environment.

**Supplementary Information:**

The online version contains supplementary material available at 10.1186/s12909-025-07237-0.

## Background

The evaluation of students’ clinical performance is an important requirement of all undergraduate medicine programmes, given the fundamental need to determine student competence [1]. Evaluation provides direction and motivation for learning, determines student progression, and upholds patient safety through identification and remediation (or removal) of those who fail to demonstrate the required competencies.

Workplace performance is regarded as the cornerstone of medical competence [2], so students are typically required to complete a series of specific clinical placements each year. The most common assessment modality used to determine satisfactory progress in these placements is a clinical supervisor evaluation [3], usually performed by senior clinicians. These evaluations often use an analytical framework to divide overall competence into domains such as knowledge and skills, which are measured discretely [2]. Formative assessment is performed to collect data which can be used to improve student learning, and to improve student performance itself, whilst summative assessment measures how much a learner retains at the completion of their learning sequence [4].

The value of an assessment is based on its utility, as described by van der Vleuten [5], whereby reliability, validity, educational impact and feasibility all shape whether a particular assessment is justifiable within a specific setting. The supervisor sign-off system to determine clinical competence is longstanding in medical education, and its use in assessment is very familiar to supervisors [6]. Potential advantages include low administration costs and time efficiency [7], provision of objectivity through standardised scoring [1], and high validity inherent in the assessment of performance in the workplace [8].

Despite widespread use, the supervisor ratings system has been criticised for being archaic and flawed in modern day education [9]. Since the advent of the supervisor sign-off system, there has been a pervasive change in the format of undergraduate clinical attachments in hospitals to promote a wider exposure to multiple different sub-speciality placements rather than a single longitudinal placement [10]. This has resulted in more numerous but shorter placements, with frequent transitions between supervisors and a subsequent reduction in contact time with each individual supervisor [11]. This change has implications for assessment validity, as supervisor ratings might be based on a single interaction, rather than the intended longitudinal assessment of competence [3].

The lack of continued interaction with one supervisor is compounded by assessor-related measurement error. Given high student numbers it is not feasible to have the same assessor for every student, so the psychometrics of assessment are dependent on inter-rater reliability, which is difficult to safeguard. Different assessors may focus on different aspects of performance [12], may not understand the level of performance they are rating [13], and may rate the same standard differently on the same rating scale [14]. Therefore, it could be argued that the advantages of rater-based clinical assessments are offset by the psychometric drawbacks.

False-positive passing outcomes may result in patient safety issues, whilst students’ lives can be altered by false negative results [15]. Thus, assessments must be psychometrically sound to justify their use in high-stakes progress decisions [16], and if an assessment tool does not add value its use should be questioned [17]. A key psychometric property of assessment is *predictive validity*; the extent to which a test can predict a learner’s future performance [18]. Tools which predict learner performance are highly prized, as targeted interventions can be implemented for struggling learners [19]. Given the widespread use of the supervisor sign-off system in undergraduate medical education, it is imperative to demonstrate its predictive validity to justify its continued role in assessment.

We set out to investigate the following questions:

1. To what extent do end-of-placement supervisor ratings of medical students predict summative examination performance?
2. Is the predictive validity of sign-off ratings mediated by placement specialty or chronology?

## Methods

### Institutional context (Imperial College London)

Third year medical students were selected as they have the longest clinical placements at our medical school. Students rotate through three different placements: ‘Medicine’, ‘Surgery’, and ‘General Practice (GP)’. Each GP placement lasts eight weeks, while each medical or surgical attachment is either eight-weeks’ duration or composed of two different four-week mini-placements. At the end of each placement, a student is ‘signed-off’ by their named consultant (attending) supervisor. On split attachments, students have a different supervisor (and sign-off) for each mini-placement. Students are pre-assigned assessors, removing the possibility of assessor selection bias [20], and all supervisors are offered webinar and in-person training on expected student standards and how to complete the sign-off form.

The sign-off form at Imperial medical school comprises multiple domains (Clinical Skills; Practical Skills; Knowledge), with ratings on a four-point ordinal scale using the categories ‘Below Expectations’, ‘Borderline’, ‘Meets Expectations’ and ‘Above Expectations’ (Appendix 1). Students must achieve either ‘Meets Expectations’ or ‘Above Expectations’ across all ratings to pass the placement, otherwise they need to undertake a remedial placement prior to being deemed eligible to sit the summative end-of-year assessment.

End-of-year summative examinations comprise of a written applied knowledge assessment and a practical Objective Structured Clinical Examination (OSCE). The written paper has 150 Single Best Answer Questions (SBAQs) divided into subsections, with 45 questions tagged to each clinical placement (medicine; surgery; GP), and 15 tagged to medical ethics and law. The 12 OSCE stations are not tagged to a specific placement as they assess integrated skills and knowledge across specialties. From a validity perspective, questions and scenarios are blueprinted to learning outcomes and the examinations are approved by external examiners.

For the written exam, the cut score is set by the Ebel method, where an expert panel determines the difficulty of each test item and its relevance to the curriculum, to produce a difficulty matrix [21]. Outcomes are based on total percentage score; there is no separate cut score for different sections. The OSCE is assessed by analytical checklist scale marking, and standard set using the borderline regression method [22].

### Data collection

Anonymised data for all 2021-22 third year medical students (*n* = 355) were obtained across three rotations (medicine; surgery; GP). Secondary data analysis was performed to assess the relationship between student end-of-placement sign-off ratings (for knowledge (K), clinical skills (CS) and practical skills (PS)) and end-of-year examination scores (written SBAQ percentage score and OSCE aggregate score across stations). For the OSCE, comparisons with all three sign-off ratings were performed for each specialty. As the written exam did not assess clinical or practical skills, SBAQ scores were only compared to knowledge sign-off ratings. Internal consistency (Cronbach’s alpha) was calculated for the SBAQ exam (0.953), OSCE (0.751), and sign-off forms (Surgery 0.885, Medicine 0.855, GP 0.842)*.* The sign-off form values are very high for a 3-part assessment but are biased upwards because the 3 components are not scored independently as they are done by the same assessor.

### Data analysis

#### Does placement order affect clinical sign-off grade awarded?

Student performance may improve throughout the year [23], therefore placement order could affect clinical ratings awarded by supervisors. Ordered chi-squared testing assessed this with clinical placement ratings converted to ordered categorical variables (Table 1). The null hypothesis was that clinical placement order did not affect grading. Where students had two supervisor reports in split placements, ratings were averaged using the mean, therefore categorical values 2, 4, and 6, represent where mean competence is graded as being in-between the categories 1, 3, and 5. This was performed so that data could be analysed independently and a like-for-like comparison was performed, as not all attachments were split.

**Table 1:** Conversion of clinical ratings to ordered categorical variables

Categorical value for Chi-squared testing	Clinical Rating sign-off grade
1	Below Expectations
2	Borderline/Below Expectations
3	Borderline
4	Meets Expectations/Borderline
5	Meets Expectations
6	Above/Meets Expectations
7	Above Expectations

Four ratings were excluded from analysis (three in GP, one in surgery) where data were incomplete.

#### Is there a correlation between sign-off domains?

Kendall’s tau-b correlation tests were performed between PS-CS, CS-K, and PS-K for each specialty. Given the use of nine statistical tests (three clinical ratings per rotation), the Holm multiple correction method was used to determine corrected p-values for statistical significance [24]. The lowest adjusted p-value for subtest significance was calculated as *p* < 0.0011 (for an overall p-value < 0.01 (Two-tailed)).

#### How do clinical ratings compare to examination scores between specialties?

Regression analysis using Ordinary Least Squares (OLS) was performed to determine the effect of increasing specialty sign-off grade on examination scores, using the categorical values shown in Table 1 as the independent variable. Results for seven students were excluded as they did not undertake end-of-year examinations. Percentage examination scores were used for statistical testing rather than pass/fail outcomes to reduce error associated with dichotomising continuous data.

#### How do examination scores compare between different placement orders?

Percentage means and standard deviations (SDs) of specialty-specific SBAQ and total OSCE scores were calculated by placement order (e.g. having a Surgery placement 1st, 2nd or 3rd ). One-way ANOVA testing was performed, with the null hypothesis that rotation order did not affect examination scores. The critical p-value required for statistical significance was 0.016. The SBAQs on medical ethics and law were not included in the analysis as there was no direct assessment of this on clinical placements.

### Ethical approval

Ethical approval was obtained from our institutional review board, Imperial College London’s Education Ethics Review Process (EERP, reference: EERP2223-060). Consent from students for use of pre-existing secondary data was not considered necessary by the EERP. The owner of the dataset was deemed to be the School of Medicine, and the intended use of data was in line with the original purpose for its collection (to better understand the reliability and validity of assessments).

## Results

### Does placement order affect clinical sign-off grade awarded?

Table 2 demonstrates ordered chi-squared test results for the analysis of clinical placement grade and placement order across specialties.

**Table 2:** Ordered Chi-squared results for clinical rating by placement order across specialties

Rotation	Chi-squared statistic	*p*-value
Medicine	Knowledge: 0.318	0.573
	Clinical Skills: 0.910	0.340
	Practical Skills: 2.819	0.093
Surgery	Knowledge: 0.992	0.319
	Clinical Skills: 1.130	0.288
	Practical Skills: 1.133	0.287
GP	Knowledge: 0.544	0.461
	Clinical Skills: 0.122	0.726
	Practical Skills: 0.001	0.979

Chi-squared calculations show placement order did not significantly impact the sign-off grade (see Appendix 2 for full tables). Calculations were completed using the most recent clinical rating for medicine and surgery in cases where there were multiple sign-offs within a placement, and again using hybrid scores, but statistical outcomes were unchanged. Given these results, data were combined across all placement orders in subsequent analyses.

### Is there a correlation between sign-off domains?

Kendall’s tau-b tests indicated strong correlations between grades awarded to students for K, PS and CS across all specialties (Table 3), with large effect sizes [25].

**Table 3:** Kendall’s tau-b correlation of PS-CS, CS-K, and PS-K

Clinical ratings	Correlation coefficient	Significance value (two-tailed)	Number (*N*)
PS-CS (GP)	0.744	< 0.0001*	351
CS-K (GP)	0.635	< 0.0001*	353
PS-K (GP)	0.587	< 0.0001*	351
PS-CS (Medicine)	0.643	< 0.0001*	352
CS-K (Medicine)	0.701	< 0.0001*	352
PS-K (Medicine)	0.594	< 0.0001*	352
PS-CS (Surgery)	0.727	< 0.0001*	351
CS-K (Surgery)	0.674	< 0.0001*	350
PS-K (Surgery)	0.717	< 0.0001*	350

*Significant result at *p* < 0.0011

1. *How do clinical placement ratings compare to examination scores between specialties?*

Table 4 shows OLS regression analysis of performance rating for each specialty between clinical knowledge ratings and specialty-specific SBAQ scores. The differences were not statistically significant when corrected for multiple comparisons (Holm correction: significance at *p* < 0.008).

**Table 4:** OLS regression of SBAQ outcome on clinical placement knowledge (K) rating

Specialty	Percentage point change in specialty SBAQ score per increased clinical rating grade for K	Lower 95% Confidence Interval	Upper 95% Confidence Interval	*r*-squared value	*p*-value
GP	+ 0.81%	− 1.27%	+ 2.89%	< 0.01	0.445
Medicine	+ 3.51%	+ 0.63%	+ 6.38%	0.02	0.017
Surgery	− 0.28%	− 3.05%	+ 2.49%	< 0.01	0.841

The same calculations were performed for OSCE data (Tables 5, 6 and 7), with significance threshold calculated at *p* < 0.008 for K and *p* < 0.016 for CS/PS. The only significant finding was a weak relationship between clinical ratings and OSCE examination results for GP ratings of CS (Table 6). The r-squared value was very low (0.02) indicating minimal educational significance. Please see appendix 3 for box & whisker plots of outcomes.

**Table 5:** OLS regression of OSCE score on clinical placement K rating

Specialty	Percentage point change in total OSCE score per increased clinical rating grade for K	Lower 95% Confidence Interval	Upper 95% Confidence Interval	*r*-squared value	*p*-value
GP	+ 1.43%	+ 0.21%	+ 2.65%	0.02	0.021
Medicine	+ 0.76%	− 0.79%	+ 2.30%	< 0.01	0.337
Surgery	+ 0.97%	− 0.55%	+ 2.49%	< 0.01	0.209

**Table 6:** OLS regression of OSCE score on clinical placement CS rating

Specialty	Percentage point change in total OSCE score per increased clinical rating grade for CS	Lower 95% Confidence Interval	Upper 95% Confidence Interval	*r*-squared value	*p*-value
GP	+ 1.76%	+ 0.39%	+ 3.15%	0.02	0.012*
Medicine	+ 0.48%	− 0.97%	+ 1.93%	< 0.01	0.516
Surgery	+ 0.77%	− 0.77%	+ 2.30%	< 0.01	0.327

*Significant result at *p* < 0.016

**Table 7:** OLS regression of OSCE score on clinical placement PS rating

Specialty	Percentage point change in total OSCE score per increased clinical rating grade for PS	Lower 95% Confidence Interval	Upper 95% Confidence Interval	*r*-squared value	*p*-value
GP	+ 0.31%	− 1.29%	+ 1.92%	< 0.01	0.702
Medicine	+ 1.00%	− 0.63%	+ 2.63%	< 0.01	0.227
Surgery	+ 0.91%	− 0.74%	+ 2.57%	< 0.01	0.279

### How do examination scores compare between placement orders?

One-way ANOVA testing (with an adjusted p-value of < 0.0125 to account for multiple tests) revealed that placement order did not statistically significantly affect examination scores (Table 8).

**Table 8:** Specialty-specific scores by placement order

Examination	ANOVA F-value	*p*-value
GP-specific SBAQs	2.94	0.054
Medicine-specific SBAQs	1.24	0.240
Surgery-specific SBAQs	4.35	0.014
Total OSCE score	1.78	0.170

## Discussion

There was little to no correlation found between supervisor clinical ratings and student examination performance. The only statistically significant correlation was between GP supervisor ratings of CS and OSCEs scores, with a very low r-squared value of 0.02, indicating a low effect size.

This low correlation could be explained by the extended timeframe between clinical ratings and end-point assessments in this study. Predictive validity evidence is often limited by timeframe between comparative assessments [26], and it is likely that many factors affected summative scores in the timeframe between placement sign-offs and end-of-year assessments. These influences can be grouped into assessor-related, learner-related, and assessment-related.

Assessor-related factors include leniency bias and fail-adversity– both cited as limitations by supervisors within the literature [8]. There were low numbers of fail grades in this study, supporting the existence of a failure-to-fail tendency inherent in assessor ratings [27].

From a learner-related perspective, feedback and reflection on performance drives learning [28], meaning students could improve their knowledge and skills following end-of-placement feedback. Additionally, students with unsatisfactory end-of-placement sign-off ratings subsequently completed remedial placements, gaining additional learning opportunities in keeping with experiential learning theory [29]. Furthermore, the sign-off form has two passing grades (‘Meets Expectations’; ‘Above Expectations’), but no tangible reward for students who attain the higher grade. Students may not strive for an excellent grade where there is no added benefit [30], and this lack of motivation to excel may flatten the regression curve between sign-off ratings and summative examination performance. Finally, student behaviours differ in the presence of an expected assessor, with ratings of competency by expected assessors resulting in more inflated grades than ratings by unexpected assessors [31], and this affects predictive validity.

Possible assessment-related factors which could contribute to a lack of significance include under-sampling of students due to a lack of contact time in short attachments, overlapping sign-off domains and single-supervisor sign-offs. This study was based at an institution which uses one assessor for clinical ratings, in contrast to the multi-assessor model used in some studies on the predictive validity of clinical ratings [31–33]. The inherently higher assessor variance in a single assessor format [7] may reduce predictive validity.

A major shortcoming in clinical assessment is the high correlation between scores in different domains of competence for individual learners, likely due to the *Halo effect* [34]. Previous investigation into this determined that assessors tend to rate all domains along only two different dimensions; medical knowledge and interpersonal skills [35], a phenomenon exacerbated by short attachments and reduced assessor-learner contact time [31]. The strong inter-domain correlation seen between clinical ratings in this study is in keeping with the literature, and may imply that CS and PS ratings could be combined.

A further assessment-related factor is that while OSCE stations were considered integrated pan-specialty assessments, some aspects of performance are less likely to be observed in routine clinical practice by particular supervisors (such as a respiratory examination by a surgical supervisor). This reduces the construct-validity in making comparisons with overall OSCE scores, and may contribute to lack of significant correlation found. Additionally, while SBAQs were tagged to one specialty, there is overlap in knowledge required.

There were no statistically significant findings within medicine or surgery cohorts, and the only statistically significant predictive validity was the correlation between GP ratings of students’ CS and OSCE scores. This can in part be explained by increased contact time, the longitudinal supervision model, and contrasting working pattern in GP versus hospital-based settings. However, with r-squared of 0.02, the effect size is likely educationally insignificant, because only 2% of variation in total OSCE scores can be explained by variation in students’ CS ratings for GP.

Ratings of PS were not statistically significantly correlated with examination results. This might be because sufficient contact time is considered necessary for an accurate assessment of learner competence [36], and consultant supervisors do not routinely observe students performing practical skills.

In terms of placement order, there was no statistical or educational difference between sign-off grades awarded at different placement times. This may indicate that supervisors adapt performance expectations with increased proximity to examinations, student performance remains relatively constant over the course, or completing a placement in one specialty does not contribute to increased performance in a later placement.

### Which changes could be considered to the sign-off process?

It could be argued that the sign-off process need not be uniform across placements. The predictive nature of CS ratings from GP supervisors in contrast to other specialties, implies that longer student-supervisor contact time improves predictive validity. However, there may also be other factors involved, as multiple additional barriers to making competency judgements exist in hospital settings. These include the more varied hospital learning environment with its frequent interruptions [28], differing levels of learners [37], and larger number of individuals who can act in supervisory roles when the allocated supervisor is not present.

Suggested changes to the sign-off process include:

- Use of multi-source feedback (MSF) in hospital settings.

In hospital settings, including the opinions of junior team members and clinical skills tutors could be considered as part of the sign-off process. Supervisors may already use several informal sources of information to aid in sign-off decisions [6], and feedback from a diverse pool of raters can supply multiple legitimate truths to the learner to aid in their development [38]. Clinical skills tutors frequently supervise students performing such skills in contrast to consultant supervisors. Additionally, junior doctors may provide more accurate evaluations of student performance [31], potentially due to their greater social and cognitive congruence with learners.

There are, however, some drawbacks to using MSF as a sign-off requirement. With multiple raters there is a risk of loss of oversight of student performance, so continuing to have a primary named supervisor could maintain accountability. Studies have indicated four-to-eleven raters are required for 80% inter-rater reliability [6, 7], but this increases institutional administration load and may reduce assessment feasibility. To ensure assessment validity, all additional supervisors would require training, and measures would need to be taken to combat assessor selection bias by pre-selecting multiple supervisors per student. Student workload and stress levels may also increase with the need to schedule multiple sign-offs.

b) Increase supervisor-learner contact time on hospital placements.

The validity of performance assessments is limited by a lack of contact time, a shortcoming more pronounced in hospital settings [39]. Brief contact can normalise snapshot competency ratings, jeopardising the utility of the assessment, which is dependent on the assessor observing a sufficient sample of student behaviours [40].

c) Embedded in-placement assessments.

A single assessment alone cannot determine competency, and multiple sampling methods improve reliability through triangulation [40]. Embedded in-placement assessments could be introduced, such as standardised SBAQs or OSCEs, or the mandatory completion of workplace-based assessments (WPBAs). These are commonly used in medical education and are familiar to both supervisors and learners [1].

A written progress examination focused on a student’s current placement would present a validated method of assessing student knowledge and could identify areas for concern [41], while the increased assessor numbers in OSCEs mitigates the large variance seen in single supervisor assessments [8]. Furthermore, assessment feedback is valuable for learners and assessment itself drives learning behaviours [1], therefore embedded in-placement assessment could stimulate student development.

A barrier to using additional assessments is feasibility. A question bank would be required for an SBAQ examination where questions would need to be continually replenished, while an OSCE would require examiner recruitment and training and the creation of stations and marking schemes. Additionally, incorporating large numbers of assessments can adversely affect educational impact because students become tick-box focused rather than learning focused [42]. Finally, as ratings tend to generalise across constructs [6], the mandatory use of WPBAs in the sign-off process may not alter student ratings.

### Limitations

Our study included data pertaining to only one medical school, and thus generalisability is limited. Other institutions may have differing sign-off processes and supervisor training methods. The use of end-of-year examinations as a benchmark for students’ knowledge and skills has its downsides as confounding factors can affect results given the distance from initial measurement to outcome.

Finally, this study investigates predictive validity with multiple variables in place (differing specialties; differing placement durations; differing numbers of assessors). It is difficult to determine whether any one of these factors pre-dominates, or indeed whether one counteracts another.

Further research could explore the differences in predictive validity with only one variable in place (differing specialties; differing placement durations; differing numbers of assessors), to identify the dominant factor. Triangulation of study population through collaboration with different institutions would aid generalisability, a prospect which will become more feasible with the introduction of a standardised national examination for medical students in the UK [43].

Furthermore, it would be worth exploring whether a pass-fail ratings scale for clinical placements shows higher predictive validity than the discriminatory scale used here.

### Implications for current practice

Determining whether a student has reached the passing standard is a fundamental outcome for the individual and the programme, and medical institutions have an ethical duty to ensure their assessments are robust and fair [44]. Given the lack of predictive validity seen for supervisor clinical ratings of medical students across a significant timeframe in this study, the current sign-off practice should be reconsidered.

Potential changes to the sign-off process can be informed by this study, and different sign-off requirements may be considered between GP and hospital-based supervisors.

## Conclusions

Since end-of-placement supervisor ratings did not demonstrate educationally significant predictive validity towards end-of-year examinations in this study, we suggest the current model of end-of-placement sign-offs requires alteration.

Multi-source feedback, embedded in-placement assessments and formalised supervision time in supervisor’s work schedules should be considered moving forwards.

## Electronic supplementary material

Below is the link to the electronic supplementary material.

Supplementary Material 1

## Data Availability

Data are provided within the manuscript and supplementary information files. Restrictions apply to the individual assessment data, which are not publicly available.

## Abbreviations

CS: Clinical Skills
EERP: Educational Ethics Review Process
K: Knowledge
GP: General Practice
MSF: Multi-source Feedback
OSCE: Objective Structured Clinical Examination
OLS: Ordinary Least Squares
PS: Practical Skills
SBAQ: Single Best Answer Question
