# Supplementary material for: What is the predictive validity of clinical placement sign-off forms for medical students?
Source: BMC Med Educ. 2025 Jun 5;25:840. doi: 10.1186/s12909-025-07237-0 (PMC12143069; doi:10.1186/s12909-025-07237-0)
Supplement: Supplementary file 1 — Supplementary Material 1 [file 12909_2025_7237_MOESM1_ESM.pdf]

APPENDICES

Appendix 1:

Sign-off grading

Please select a grade for each domain and add a comment

Clinical Skills *
Has the student achieved an appropriate level of skill in history taking/examination/formulation of differential diagnoses deemed appropriate for their current year of study?

- Above Expectations
- Meets Expectations
- Borderline
- Below Expectations
- Unable to Comment

Comments

Practical Skills *
Has the student demonstrated adequate levels of competence, in examination and practical skills, deemed appropriate for their current year of study? This includes completion of DOPS/DOCS.

Comments

Knowledge *
Has the student applied an appropriate level of knowledge (biomedical, psychological, social, ethical) and demonstrated reflective practice, deemed appropriate for the current year of study?

Comments

Appendix 2:

Clinical ratings by placement order

Table a: Clinical rating frequency by GP placement order

Clinical Rating	Number of Ratings by Placement Order (Knowledge)			Number of Ratings by Placement Order (Clinical Skills)			Number of Ratings by Placement Order (Practical Skills)		
	1	2	3	1	2	3	1	2	3
Above Expectations	26	35	27	18	33	18	14	22	14
Meets Expectations	88	82	89	99	84	99	103	95	103
Borderline	5	0	1	2	0	0	0	0	0
Below Expectations	0	0	0	0	0	0	0	0	0
Total	119	117	117	119	117	117	117	117	117
	Chi-squared statistic†: 0.544			Chi-squared statistic†: 0.122			Chi-squared statistic†: 0.001		
	p-value: 0.461			p-value: 0.726			p-value: 0.979		

Table b: Clinical rating frequency by medicine placement order

Clinical Rating	Number of Ratings by Placement Order (Knowledge)			Number of Ratings by Placement Order (Clinical Skills)			Number of Ratings by Placement Order (Practical Skills)		
	1	2	3	1	2	3	1	2	3
Above Expectations	13	17	15	17	17	16	13	12	11
Above/Meets Expectations	39	41	34	33	42	31	30	35	21
Meets Expectations	64	60	69	66	59	71	73	70	85
Meets Expectations / Borderline	0	0	1	0	0	1	0	1	3
Borderline	0	0	1	0	0	0	0	0	0
Borderline/ Below Expectations	0	0	0	0	0	0	0	0	0
Below Expectations	0	0	0	0	0	1	0	0	0
Total	116	118	120	116	118	120	116	118	120
	Chi-squared statistic†: 0.318			Chi-squared statistic†: 0.910			Chi-squared statistic†: 2.819		
	p-value: 0.573			p-value: 0.340			p-value: 0.093		

Table c: Clinical rating frequency by surgery placement order

Clinical Rating	Number of Ratings by Placement Order (Knowledge)			Number of Ratings by Placement Order (Clinical Skills)			Number of Ratings by Placement Order (Practical Skills)		
	1	2	3	1	2	3	1	2	3
Above Expectations	12	22	16	14	19	16	9	19	14
Above/Meets Expectations	6	17	12	6	16	13	6	14	8
Meets Expectations	98	76	89	95	81	90	101	83	97
Meets Expectations / Borderline	0	1	0	0	0	0	0	0	0
Borderline	0	0	1	1	0	0	0	0	0
Borderline/ Below Expectations	0	0	0	0	0	0	0	0	0
Below Expectations	0	0	0	0	0	0	0	0	0
Total	116	116	118	116	116	119	116	116	119
	Chi-squared statistic†: 0.992			Chi-squared statistic†: 1.130			Chi-squared statistic†: 1.133		
	p-value: 0.319			p-value: 0.288			p-value: 0.287		

†Degrees of freedom = 1 (for all tests). N < 5 in some cells reduces test results reliability.

Appendix 3:
Surgery Box & Whisker plots of clinical ratings versus exam scores

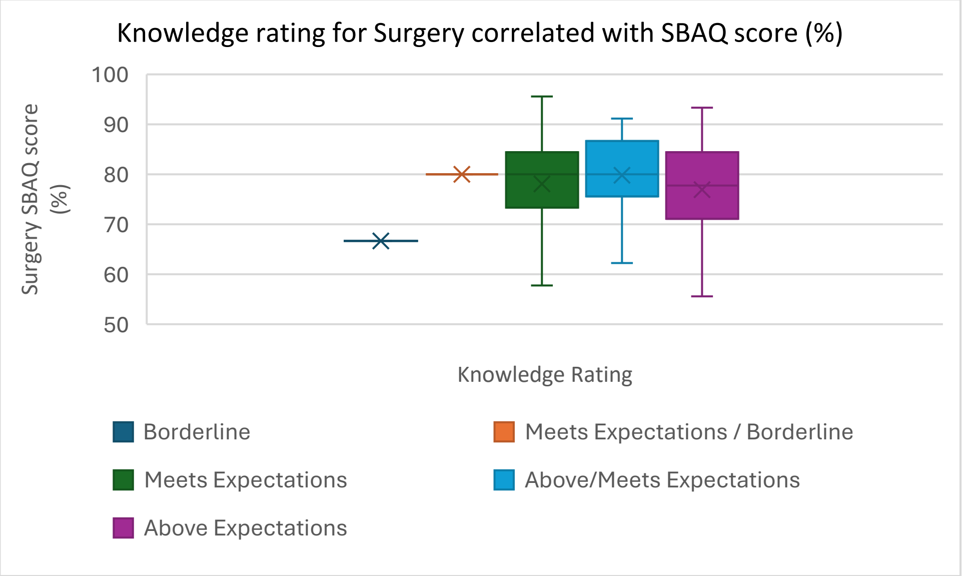

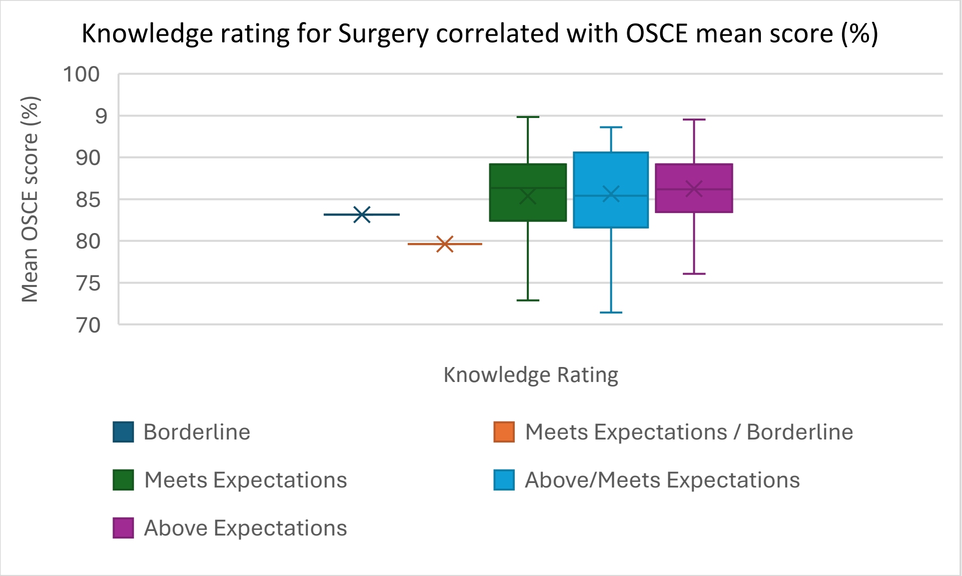

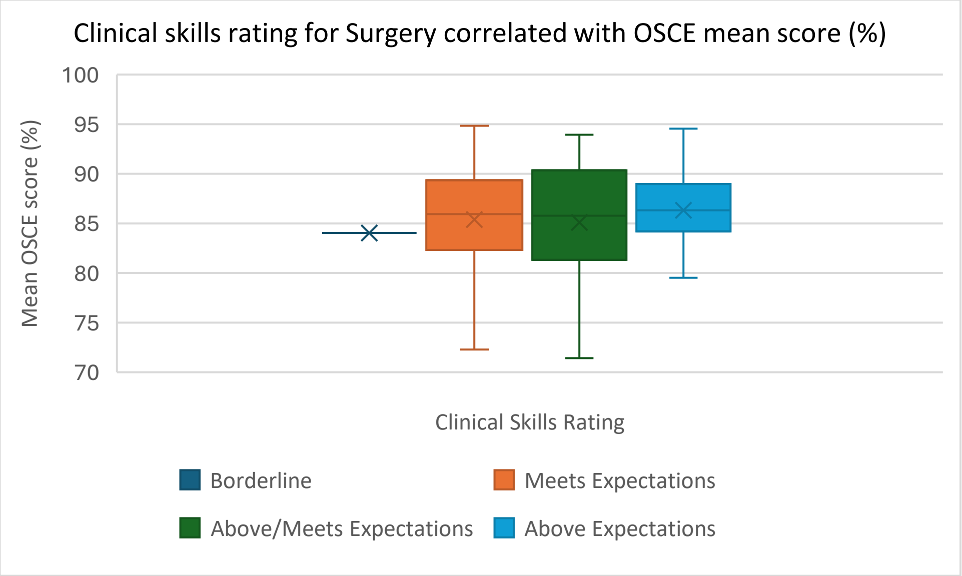
Clinical skills rating for Surgery correlated with OSCE mean score (%)
100
95
90
85
80
75
70
Mean OSCE score (%)
Clinical Skills Rating
Borderline
Meets Expectations
Above/Meets Expectations
Above Expectations

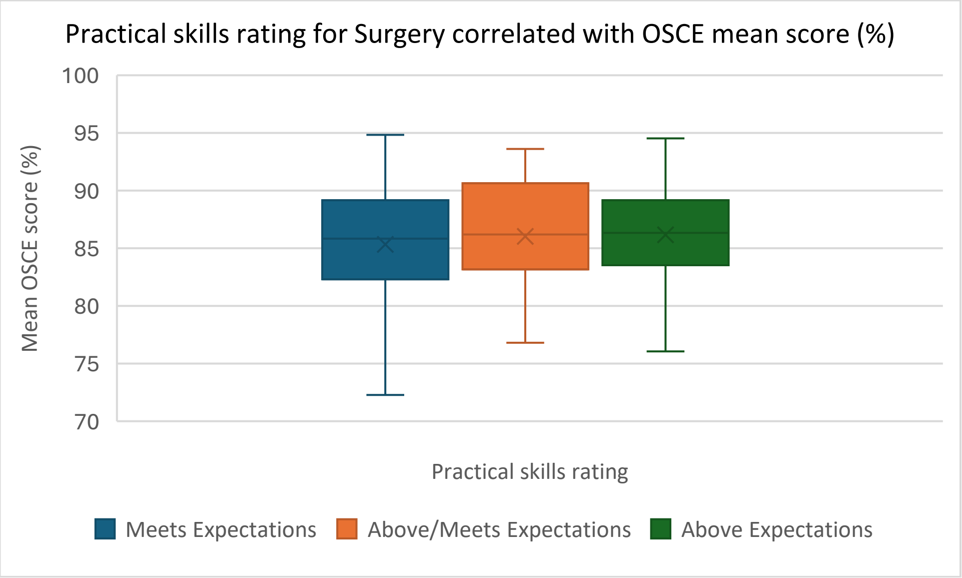
Practical skills rating for Surgery correlated with OSCE mean score (%)
100
95
90
85
80
75
70
Mean OSCE score (%)
Practical skills rating
Meets Expectations
Above/Meets Expectations
Above Expectations

Medicine Box & Whisker plots of clinical ratings versus exam scores

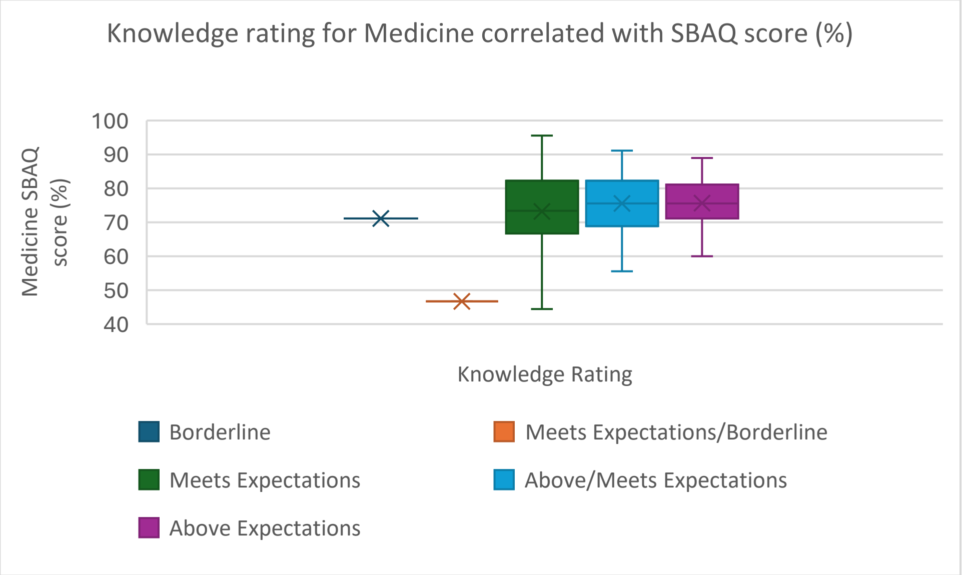

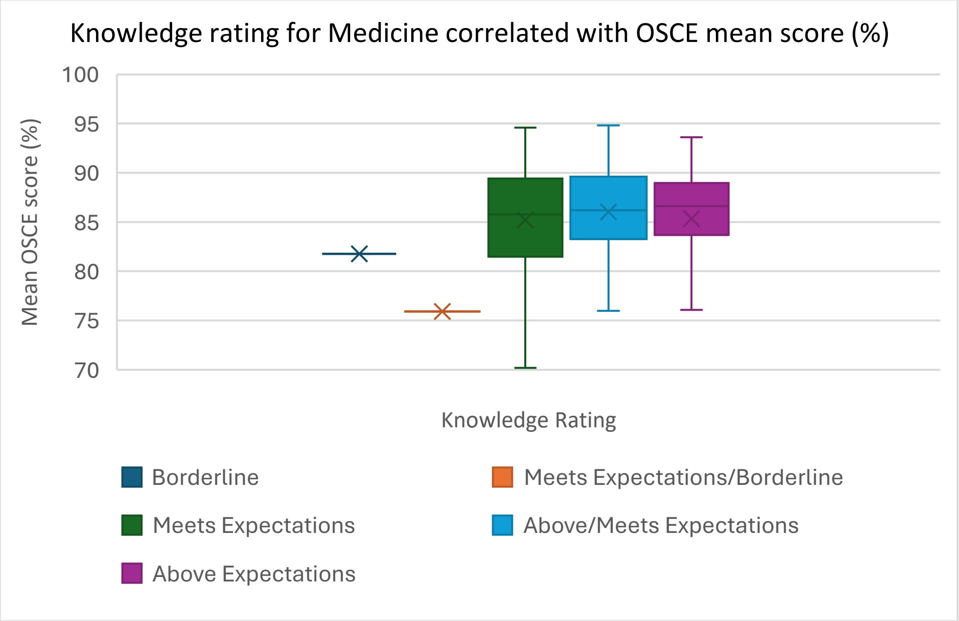

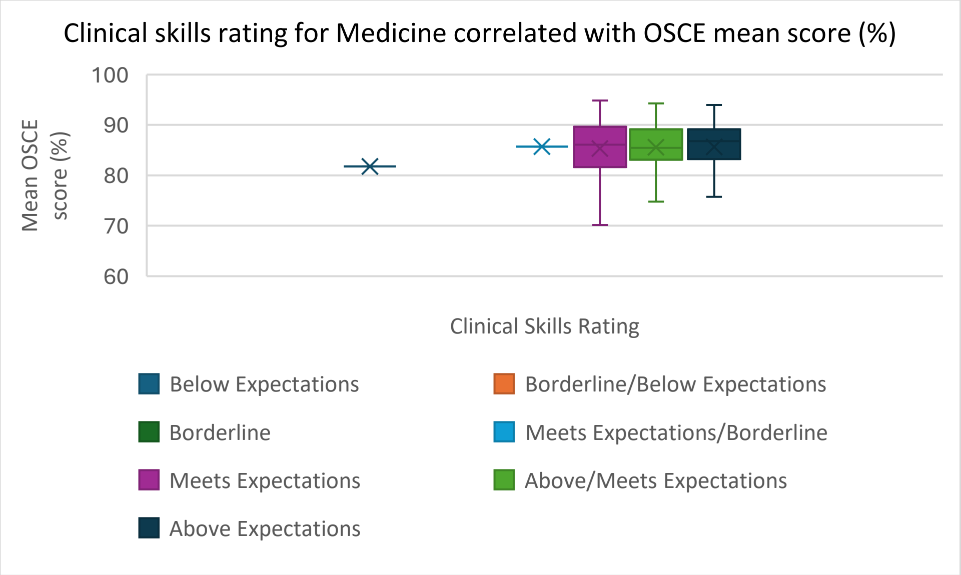
Clinical skills rating for Medicine correlated with OSCE mean score (%)
Mean OSCE score (%)
100
90
80
70
60
Clinical Skills Rating
Below Expectations
Borderline/Below Expectations
Borderline
Meets Expectations/Borderline
Meets Expectations
Above/Meets Expectations
Above Expectations

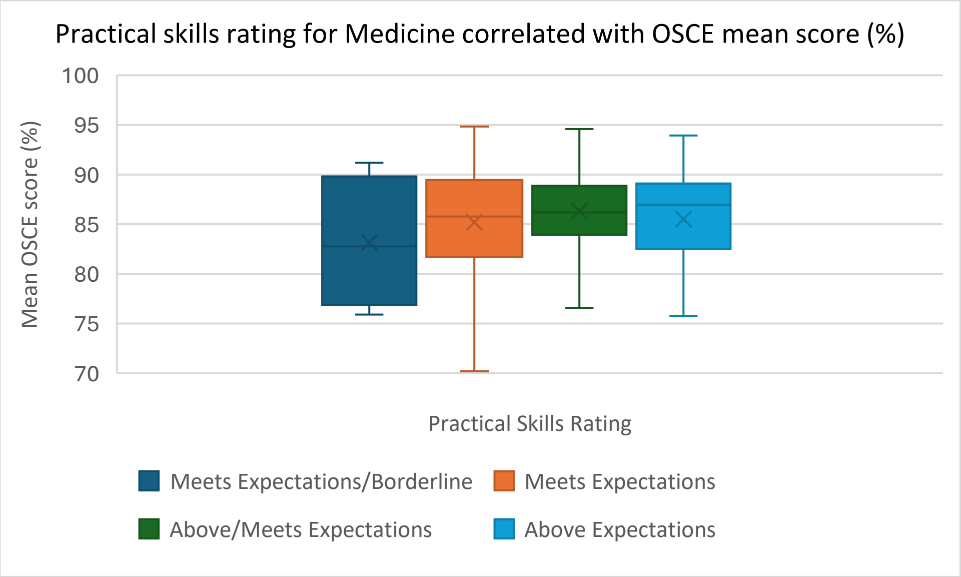
Practical skills rating for Medicine correlated with OSCE mean score (%)
Mean OSCE score (%)
100
95
90
85
80
75
70
Practical Skills Rating
Meets Expectations/Borderline
Meets Expectations
Above/Meets Expectations
Above Expectations

GP Box & Whisker plots of clinical ratings versus exam scores

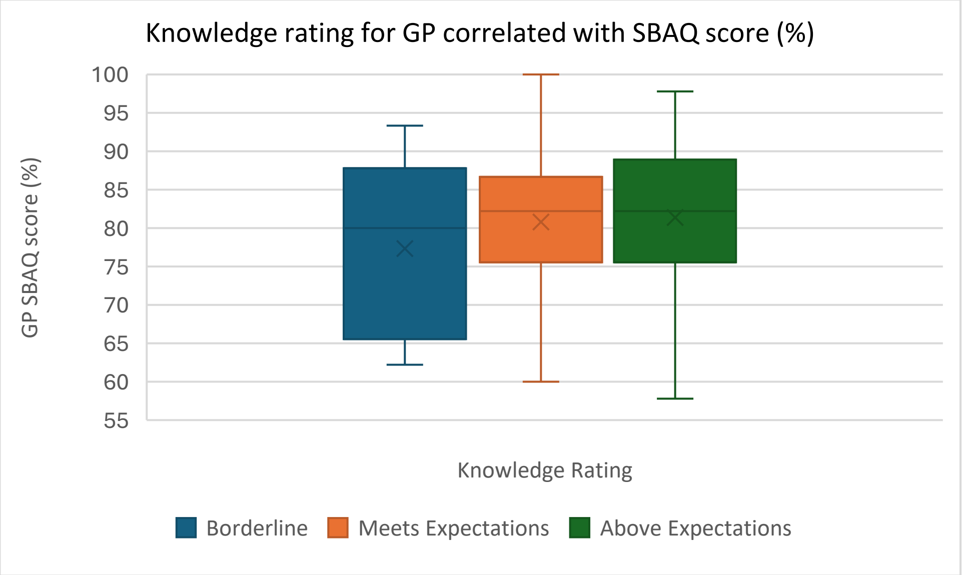

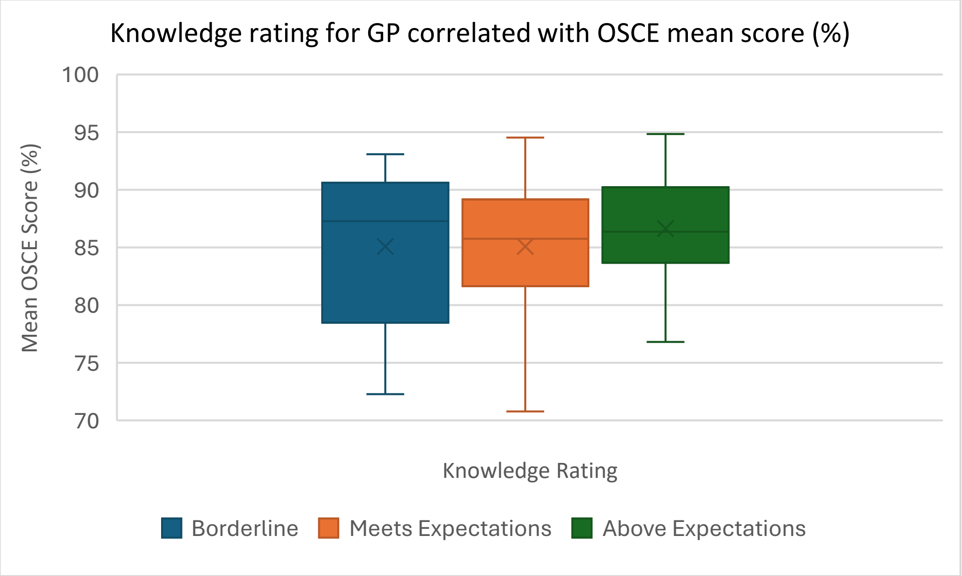

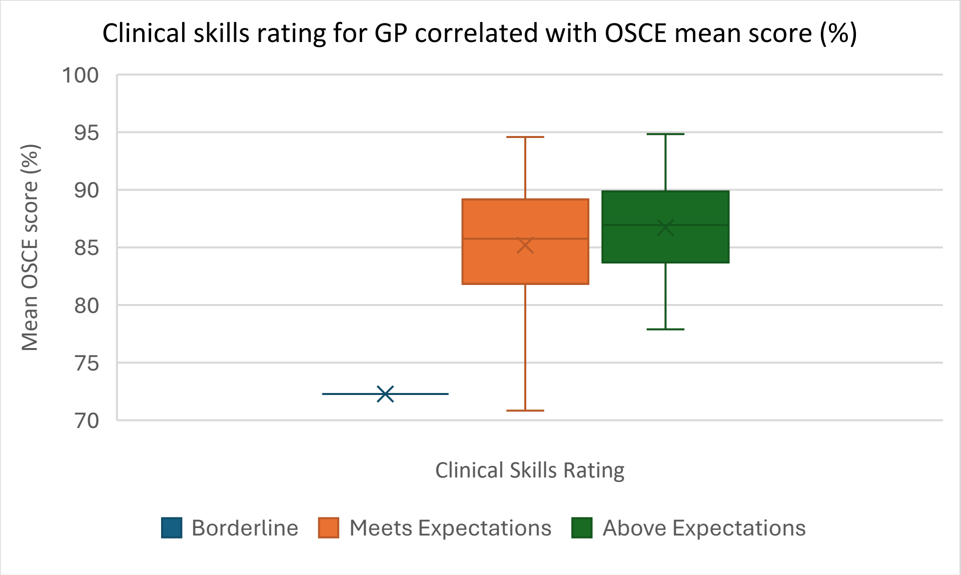
Clinical skills rating for GP correlated with OSCE mean score (%)
100
95
90
85
80
75
70
Mean OSCE score (%)
Clinical Skills Rating
Borderline
Meets Expectations
Above Expectations

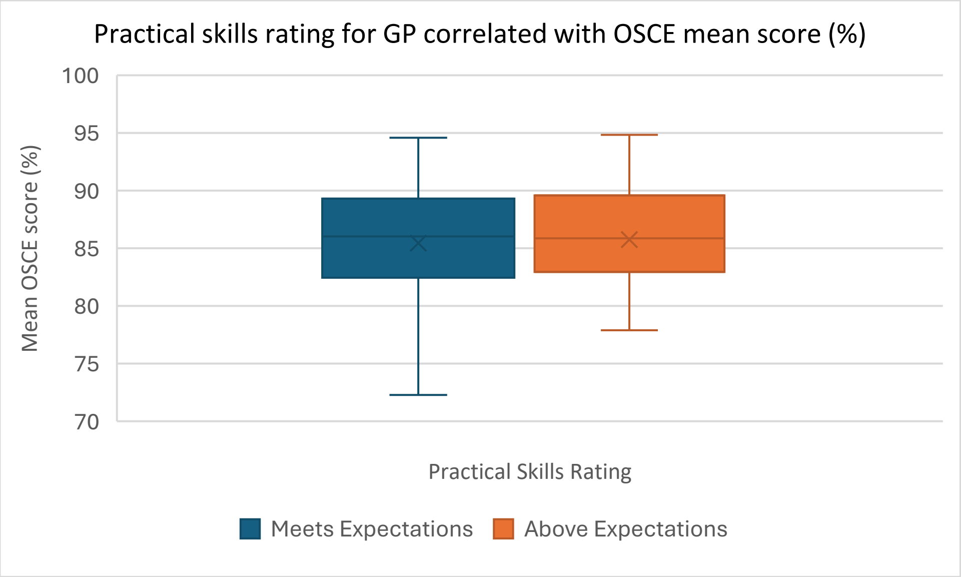
Practical skills rating for GP correlated with OSCE mean score (%)
100
95
90
85
80
75
70
Mean OSCE score (%)
Practical Skills Rating
Meets Expectations
Above Expectations
